# Cytotoxic Terpene Quinones from Marine Sponges

**DOI:** 10.3390/md8122849

**Published:** 2010-11-24

**Authors:** Marina Gordaliza

**Affiliations:** Department of Pharmaceutical Chemistry, Pharmacy Faculty, Salamanca University, Campus Miguel de Unamuno, 37007 Salamanca, Spain; E-Mail: mliza@usal.es; Tel.: +34-923-294-528; Fax: +34-923-294-515

**Keywords:** terpene quinone, terpene hydroquinone, avarone, avarol, cytotoxicity

## Abstract

The 1,4-benzoquinone moiety is a common structural feature in a large number of compounds that have received considerable attention owing to their broad spectrum of biological activities. The cytotoxic and antiproliferative properties of many natural sesquiterpene quinones and hydroquinones from sponges of the order Dictyoceratida, such as avarol, avarone, illimaquinone, nakijiquinone and bolinaquinone, offer promising opportunities for the development of new antitumor agents. The present review summarizes the structure and cytotoxicity of natural terpenequinones/hydroquinones and their bioactive analogues and derivatives.

## 1. Introduction

The few therapeutic novelties of synthetic origin that have appeared on the pharmaceutical market, of which more than 88% are based on preexisting structures, have flooded the pharmaceutical world with very similar products. This has led to a new and enthusiastic retrospective appraisal of the active principles of the molecules integrating medicinal plants and different natural sources. It is for these reasons that research on natural compounds has aroused considerable interest at the academic, commercial and governmental levels.

Of the different natural sources, the sea has become an important basis for the collection of natural compounds of use to humans and it is clear that this will continue to be the case in the future [1–13]. Interest in marine organisms, both animals and plants, as sources of active substances was boosted at the beginning of the seventies by the work of several marine research teams: Universities of Hawaii and Oklahoma and The Scripps Institution of Oceanography in California, as well as other teams in Japan and Europe. This began with the isolation of prostaglandins from corals, followed by the discovery of other derivatives. In recent years, it has been possible to isolate and characterize thousands of compounds, many of which exert important activities in several biological systems [14].

The discovery of drugs from marine natural products has enjoyed a renaissance in the past few years [15–19]. Ziconotide (Prialt^®^; Elan Pharmaceuticals), a peptide originally discovered in a tropical cone snail, was the first marine-derived compound to be approved in the United States in December 2004 for the treatment of pain [20]. Then, in October 2007, trabectedin (Yondelis^®^; PharmaMar) became the first marine anticancer drug to be approved in the European Union [21]. In a recent congress held by the American Society of Oncology (Chicago, June 2010), attention was drawn to the antitumor properties of eribulin mesylate (E7389), designed by the Japanese laboratory Eisai (Eisai Research Institute, Andover, MA, USA) for the treatment of breast cancer. This is a synthetic analogue of the natural product halichondrin B, isolated from *Halichondria okadai (Lissodendoryx* sp.), a marine sponge commonly found in Japanese seas; its antitumor activity was discovered in 1986. Eribulin binds to the vinca domain of tubulin and inhibits the polymerization of tubulin and the assembly of microtubules, resulting in the inhibition of mitotic spindle assembly, the induction of cell cycle arrest at G2/M, and, potentially, tumor regression. Eribulin mesylate is now in phase II clinical trials and is active in metastatic or locally advanced breast cancer [22,23].

In particular, the cytotoxic and antiproliferative properties of many natural sesquiterpene quinones and hydroquinones from sponges of the order Dictyoceratida [24] such as avarol, avarone, illimaquinone, nakijiquinone and bolinaquinone offer promising opportunities for the development of new antitumor agents [25,26]. This has sparked interest in the chemical composition and cytotoxicity of a large number of marine species that contain metabolites with hybrid structures between terpenes and quinones/hydroquinones. Related to such terpenequinone structures, several studies have been published addressing the chemistry, activity and mechanisms of action of the compounds [27–33].

The present review addresses the terpenylquinones of marine origin with cytotoxic properties that are active against different tumor cell lines. It also deals with the cytotoxic hydroquinones and some semisynthetic analogues of bioactive terpenequinones. The compounds described herein are mainly natural sesquiterpenes and share a decalin structure, bound via a 1C carbon chain to a ring of differently functionalized *p*-benzoquinone or hydroquinone. Cytotoxic terpenequinone/hydroquinones are usually metabolites that incorporate a bicyclic sesquiterpene unit coupled to a quinone or quinol. Most of these possess a drimane or rearranged drimane skeleton. In the present work they have been grouped on the basis of the structural resemblance of the bicyclic moiety with some diterpene skeletons.

## 2. Terpenylquinones with a Clerodane-Type Decalin Ring

**Figure 1:**
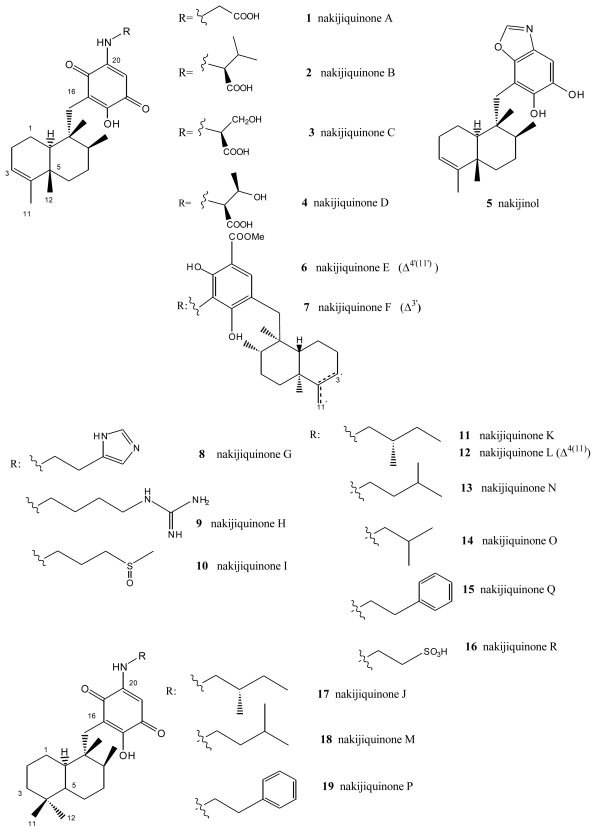
Nakijiquinones.

From extracts of sponges from the family *Spongiidae*, collected in Okinawa, it has been possible to isolate several compounds with an amino acid moiety on the benzoquinone ring: nakijiquinones A (**1**), B (**2**), C (**3**) and D (**4**) [34–36], or with a benzoxazol moiety: nakijinol (**5**) (Figure 1). It should be noted that the presence of aminoquinone compounds is not very common in natural products, these substances being the first examples of sesquiterpenequinones of natural origin with an amino acid or heterocyclic moiety. Nakijiquinones A (**1**), B (**2**), C (**3**) and D (**4**) showed *in vitro* cytotoxicity against L-1210 (IC_50_ values between 2.8 and 8.1 μg/mL) and KB (IC_50_ values between 1.2 and 7.6 μg/mL).

The new dimeric sesquiterpenoid quinones nakijiquinones E (**6**) and F (**7**) were isolated from an Okinawan marine sponge [37]. These nakijiquinones were the first dimeric sesquiterpenoid quinones possessing a 3-aminobenzoate moiety. Nakijiquinones G–I (**8**–**10**), containing a different amino group derived from amino acids, were isolated from Okinawan marine sponges of the family Spongiidae, and showed modest cytotoxicity and inhibitory activity against HER2 kinase, while nakijiquinone H (**9**) exhibited antimicrobial activity [38].

Nakijiquinones J–R (**17**, **11**, **12**, **18**, **13**, **14**, **19** and **16**), at 1 mM were tested for inhibitory activities against EGFR and HER2 tyrosine kinases. Among them, nakijiquinones P (**19**) and R (**16**) exhibited inhibitory activities against EGFR (76 and >99% inhibition, respectively), while nakijiquinones N (**13**), O (**14**) and R (**16**) showed inhibitory activities against HER2 (66%, 59% and 52% inhibition, respectively) [39]. The HER2/Neu tyrosine kinase receptor is hugely overexpressed in about 30% of primary breast, ovary, and gastric carcinomas. Nakijiquinones are the only naturally occurring inhibitors of this important oncogene, and structural analogues of nakijiquinones may display inhibitory properties against other tyrosine kinase receptors involved in cell signaling and proliferation [40].

**Figure 2:**
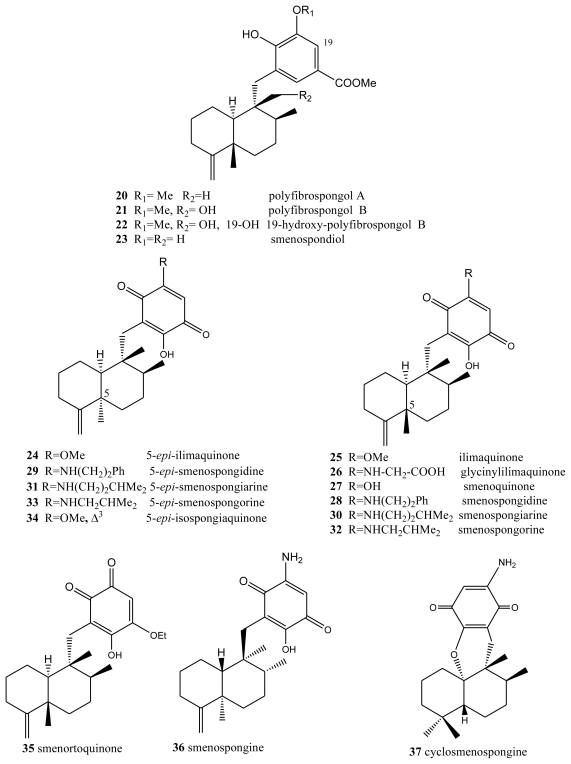
Polyfibrospongols, ilimaquinones, smenospongines and related compounds.

Another type of compound with bioactive properties includes those whose benzo(hydro)quinone ring is substituted by a methoxycarbonyl group, as is the case of polyfibrospongols and smenospondiol. Extracts of the marine sponge *Polyfibrospongia australis*, collected in Taiwan, were used to isolate polyfibrospongols A (**20**) and B (**21**) (Figure 2) [41], compounds showing cytotoxic activity against different tumor lines. From the South China sponge *Dysidea arenaria*, a new sesquiterpenoid hydroquinone, 19-hydroxy-polyfibrospongol B (**22**), was isolated, along with polyfibrospongol B (**21**) and other known terpenequinones [42].

Also showing cytotoxic, as well as antibacterial, activity is smenospondiol (**23**), isolated from dichloromethane extracts of several species of the genus *Smenospongia* and with a very similar structure [43,44]. This compound has also been called dictyoceratin A by other authors [45]. These compounds showed interesting levels of cytotoxicity when assayed against the P-388 (mouse lymphoma), KB-16 (human nasopharyngeal carcinoma) and A-549 (human lung carcinoma) cells, their CI_50_ values lying between 0.6 and 2.0 μg/mL.

5-*epi*-Ilimaquinone (**24**) showed cytotoxic activity (IC_50_) against P-388 leukemia cells (2.2 μg/mL) and different solid tumors: A-549 (0.9 μg/mL), HT-29 (3.4 μg/mL) and B16/F10 (1.1 μg/mL) [46]. It has been suggested that ilimaquinone (**25**) induces a concentration-dependent antiproliferative effect in several types of cancer cell lines, including PC-3 and LNCaP prostate cancer, A549 non-small cell lung cancer and Hep3B hepatocellular carcinoma cells. The anticancer mechanism of ilimaquinone in the representative PC-3 cells was identified. Ilimaquinone (**25**) induced a time-dependent increase in G1 phase arrest and a subsequent increase in the hypodiploid sub-G1 phase (apoptosis) of the cell cycle. The cell cycle arrest was associated with a sustained high level of nuclear cyclin E but the absence of DNA synthesis, according to flow cytometric analysis, indicated an incomplete S phase. Although ilimaquinone-induced Golgi vesiculation, the data showed that the inhibition of cancer cell growth did not occur through Golgi fragmentation. Ilimaquinone did not activate extracellular signal-regulated kinase and phosphatidylinositol 3-kinase but induced an up-regulation, nuclear translocation and gene 153-induced DNA damage (CHOP/GADD153). Furthermore, the ilimaquinone-mediated antiproliferative effect was significantly reduced in antisense CHOP/GADD153-overexpressing cells. Ilimaquinone (**25**) also inhibited the DNA binding of NF-κB; however, this inhibitory effect cannot explain the ilimaquinone-induced anticancer effect. In brief, it is suggested that ilimaquinone (**25**) induces its antiproliferative effect through the G1 arrest of the cell cycle and the up-regulation and nuclear translocation of CHOP/GADD153 [32]. Bioassay-guided isolation from the marine sponge *Hippospongia* sp., collected at Palau, led to the isolation of three sesquiterpene quinone metabolites: ilimaquinone (**25**), 5-*epi*-ilimaquinone (**24**), and 5-*epi*-isospongiaquinone (**34**) (Figure 2). The cytotoxicity against the NCI-H460, HepG2, SF-268, MCF-7, HeLa, and HL-60 human tumor cell lines, the inhibitory effects on the maturation of starfish oocytes, and cell cycle arrest in the HepG2 cell line were evaluated [47].

One compound closely linked to those above is glycinylilimaquinone (**26**), a metabolite isolated from a specimen from the genus *Fasciosponia* from the Phillipines, its structure having being determined by spectroscopic analysis and chemical synthesis [48,49]. This structure displayed cytotoxic activity, with IC_50_ = 7.8 μg/mL, against a human carcinoma tumor cell line (HT-29). However, when it was tested against P-388 mouse lymphoma *in vivo*, the maximum dose tolerated did not display cytotoxic activity.

Other compounds with free hydroxyl and/or amino functions on the benzo(hydro)quinone ring have been isolated from the genus *Smenospongia*. Bioassays performed on the dichloromethane extract of different species of this genus revealed both cytotoxic and antibacterial activities. From this extract, smenosquinone (**27**), smenospongidine (**28**), smenospongiarine (**30**), smenospongorine (**32**) and smenorthoquinone (**35**) were isolated [50]. The cytotoxicity of some of these compounds was assessed against L.1210 leukemia cells. The results for smenoquinone, smenospongiarin and smenortoquinone were IC_50_: 2.5, 4.0 and 1.5 μg/mL, respectively.

The methanol-chloroform extract of several species of the genus *Smenospongia* also afforded smenospongin (**36**), from the enantiomeric series with respect to the above described compounds. Smenospongin (**36**), which has also been isolated from *Dactylospongia elegans*, showed promising biological activities: cytotoxicity against L-1210 leukemia cells with a LD_50_ of 1 μg/mL. Smenospongine (**36**) induced erythroid differentiation and G1 phase arrest of K562 chronic myelogenous leukemia cells. In that study, the effect of smenospongine (**36**) on the cell cycles of other leukemia cells, including HL60 human acute promyelocytic leukemia cells and U937 human histiocytic lymphoma cells, was investigated by flow cytometric analysis. Smenospongine (**36**) induced dose-dependent apoptosis in HL60 and U937 cells. Smenospongine (**36**) treatment increased the expression of p21 and inhibited the phosphorylation of Rb in K562 cells, suggesting the p21-Rb pathway plays an important role in G1 arrest in K562 cells. However, based on a luciferase assay using transfected K562 cells, the p21 promoter was not activated by smenospongine (**36**) treatment. Smenospongine might induce p21 expression via a mechanism other than the transactivation of the p21 promoter [51].

*D. elegans*, from Papua Nueva Guinea and Thailand, contains a total of 17 merosesquiterpenoids, among which are (+)-*epi*-smenospongiarin (**31**) and (+)-*epi*-smenospongidin (**29**) [46]. These compounds were assayed *in vitro* against solid tumor models (A-549, HT-29 and B16/F10) and leukemia cells (P-388), (+)-*epi*-smenospongiarin (**31**), with IC_50_ values between 0.6 and 0.9 μg/mL being of particular interest.

A new sesquiterpene aminoquinone, 5-*epi*-smenospongorine (**33**), together with nine known sesquiterpene quinone/phenols, were isolated from the marine sponge *Dactylospongia elegans*. The structure-activity relationship study of these compounds revealed that the quinone skeleton is indispensable and the amino group plays an important role for their differentiation-inducing activity to K562 cells into erythroblast [52]. The new sesquiterpenoid aminoquinone, cyclosmenospongine (**37**), containing a dihydropyran ring, was isolated from an Australian marine sponge *Spongia* sp., along with the known metabolites, smenospongiarine (**30**), ilimaquinone (**25**) and smenospongine (**36**) [53].

A unified synthesis of several quinone sesquiterpenes is described by Ling *et al.* [54].

**Figure 3:**
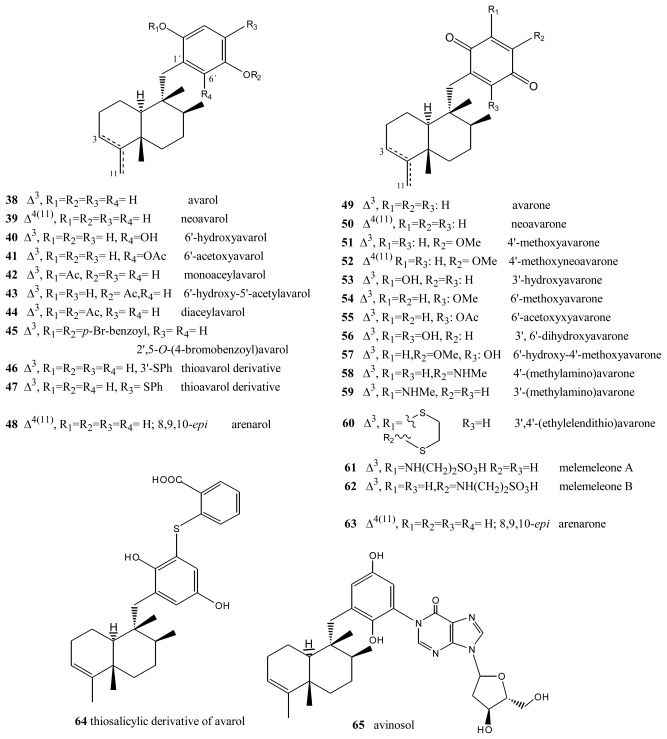
Avarols and avarones.

Avarol (**38**), a sesquiterpene hydroquinone, and its quinone derivative avarone (**49**) (Figure 3) are secondary metabolites isolated from the marine sponge *Dysidea avara*. Both compounds were first discovered as anti-leukemia agents *in vitro* and *in vivo*, and later it was found that they had an *in vitro* inhibitory capacity against HIV-1 [55–60]. Controlled clinical studies revealed, however, that it was not efficient in the clinical treatment of patients with AIDS. Additionally, the potent T-lymphotropic cytostatic activity shown by avarol (**38**), and its low toxicity in mice, its ability to cross the blood-brain barrier and its ability to stimulate the synthesis of interferon make both these compounds optimum candidates for transformations aimed at improving their cytostatic and antiviral activity [12–17,55–60].

The first avarone analogues were obtained by semisynthesis. Among the main substitutions are those performed on the quinone ring, including hydroxyl, methylamino, ethylamino, and glucosamine groups and different essential amino acids at positions 3′ or 4′ [56,61]. Cytostatic activity was assayed by analyzing the capacity of these compounds to inhibit the growth of fibroblasts, lymphocytic leukemia and lymphoblastic B and T cells. Avarol (**38**) and avarone (**49**) showed very similar inhibitory activity against the cell lines assayed (IC_50_ = 13.9–15.6 μM), as did the methyl, ethyl and glucosylaminated analogues and the alaninyl, phenylalaninyl and leucinyl derivatives. By contrast, the serinyl and cysteinyl derivatives were significantly less active. Antiviral activity was evaluated against the following viral types: HIV-1, ASFV, HSV-1, HSV-2, polio and VSV. Because of its activity (IC_50_ = 0.04 μM HSV-1 and 0.2 μM HSV-2), acyclovir is used like control compound against herpes simplex. Avarone (**49**) showed a more potent activity against HIV-1 (IC_50_ = 1.5 μM) than avarol (**38**) (IC_50_ = 2.9 μM) *in vitro.* Among the derivatives of avarone, modification in the quinone ring always afforded a loss of anti-HIV-1 potential, with the exception of leucinyl- and cysteicyl-avarone, which were as potent as avarone (**49**), the latter derivative being even more selective.

All the derivatives selectively inhibited polio virus, but were almost completely inactive against the other viruses assayed. Against polio virus, avarol (**38**), avarone (**49**) and the 3′-methyl and 3′-ethyl aminoderivatives were more potent and selective inhibitors. The 3′-substituted analogues maintained potency and selectivity, while the 4′-substituted analogues showed a significantly lower potency and in some cases selectivity, the only exceptions being the 4′serinyl and 4′-cysteinyl derivatives of avarone.

Different derivatives of avarol and avarone have been isolated from other species of the genus *Dysidea*. These included, neoavarol (**39**), neoavarone (**50**), 4′-methoxyavarone (**51**) and 4′-methoxyneoavarone (**52**) isolated from a specimen in Okinawa [62], while from the extract of *Dysidae cinera* (collected in the Red Sea), 6′-hydroxyavarol (**40**), 6′-acetoxyavarol (**41**), 3′-hydroxyavarone (**53**), 6′-acetoxyavarone (**55**), 3′,6′-dihydroxyavarone (**56**) and 6′-hydroxy-4′-methoxyavarone (**57**) were isolated [63]. Some of these compounds showed cytotoxic, antimicrobial and anti-HIV properties. The results of cytotoxicity assays against P-388 mouse lymphoma indicated high potency for 3′-hydroxyavarone (**53**), 6′-acetoxyavarol (**41**) and 3,6′-dihydroxyavarone (**56**), with IC_50_ values of 0.6, <0.6 and 1.2 μg/mL, respectively.

Additionally, and related to the above, from different extracts of *Dysidea avara* collected from different places (Japan, the Solomon Islands, and others), minor metabolites, analogues of avarol and avarone, were isolated: monoacetylavarol (**42**), diacetylavarol (**44**), 6′-hydroxy-5′-acetylavarol (**43**), 4′-methylaminoavarone (**58**), melemeleone A (**61**) and melemeleone B (**62**). These substances were subjected to different biological activity assays both with regard to their cytotoxicity and their capacity for enzyme inhibition [54,64–66]. The cytotoxicity assays performed for diacetylalvarol (**44**) revealed levels comparable with those of avarol (**38**), both in tests with *Artemia salina* (avarol LD_50_ = 0.18 ppm; diacetylavarol, LD_50_ = 0.15 ppm) and potato disk assays (avarol, 64% inhibition; diacetylavarol, 55% inhibition). Regarding the values for enzyme inhibition, only melemeleone B (**62**) proved to have a certain activity against PTK pp60^v-sarc^ (dose: 20 μg/mL) with an IC_50_ = 28 μM.

Nine alkyl(aryl)thio derivatives of avarone were synthesized by nucleophilic addition of thiols or thiophenol to avarone, and their cytotoxicity was compared that of aminoavarones. Most derivatives showed cytotoxic activity against tumor cell lines (human cervical carcinoma, HeLa cells, human melanoma Fem-X and human leukemia K-562), with IC_50_ values lower than 10 μM for some of these, in particular those with electron-donating substituents. Most compounds showed activity against all three cell lines, but leukemia cells were generally the most susceptible, with IC_50_ values similar to cisplatin for some methylamino and methoxyavarone derivatives. The exceptions were 4′-(methylamino)avarone (**58**) and 3′,4′-(ethylenedithio)avarone (**60**), which were more active against melanoma cells, although overall the latter compound showed low activity. The most active compound was 4′-(methylamino)avarone (**58**), with an IC_50_ value of 2.4 μM against melanoma Fem-X cells, and no cytotoxicity against normal lymphocytes [31]. A highly efficient total synthesis of (+)-avarone, (+)-avarol, (−)-neoavarone, (−)-neoavarol and (+)-aureol has been achieved [67]. An *in vitro* cytotoxicity assay against U937 human histiocytic lymphoma cells determined the order of cytotoxic potency ((−)-neoavarone > (+)-avarone > (+)-aureol > (+)-avarol > (−)-neoavarol) and some aspects of their structure-activity relationships [67].

Upon acylation, avarol (**38**) afforded several compounds, two of them— 2′,5′-*O*-(4-bromobenzoyl)avarol (**45**) and diacetylavarol (**44**)—showed cytotoxicity against Hepa (human hepatoma) and KB cell lines, respectively [68]. The semisynthesis of 13 new thioavarol derivatives and an *in vitro* evaluation of the photodamage response induced by UVB irradiation were described. The ability of the thioavarol derivatives prepared to inhibit NF-κB activation and TNF-α generation in HaCaT cells, as well as their antioxidant capacity in human neutrophils, was also studied. The two monophenyl thioavarol derivatives **46** and **47** lacked cytotoxicity and were considered promising UVB photoprotective agents owing to their potent inhibition of NF-κB activation, with a mild antioxidant pharmacological profile [69]. A thiosalicylic derivative **64** of avarol was found to be a potent inhibitor of superoxide generation in human neutrophils, and it also potently inhibited PGE_2_ generation in the HaCaT human keratinocyte cell line [70]. 3′-methylaminoavarone (**59**) had the best antiproliferative profile, owing to its inhibition of 3H-thymidine incorporation into HaCaT cells, with a potency similar to the reference compound anthralin [70]. Avinosol (**65**), a new merotepenoid isolated from the marine sponge *Dysidea* sp. collected in Papua New Guinea, appeared to be the first example of a naturally occurring meroterpenoid-nucleoside conjugate, and showed anti-invasion activity in cell-based assays [71].

Two sesquiterpenoids with a quinone and hydroquinone moiety, respectively, were isolated from the marine sponge *Dysidea arenaria*: arenarol (**48**) and arenarone (**63**). These compounds showed cytotoxic activity when assayed against P-388 leukemia cells, with ED_50_ = 17.5 μg/mL for arenarol (**48**) and ED_50_ = 1.7 μg/mL for arenarone (**63**) [72]. Arenarol (**48**) showed DPPH radical scavenging activity with an IC_50_ value of 19 μM [73].

## 3. Terpenylquinones with a Labdane-Type Decalin Ring

**Figure 4:**
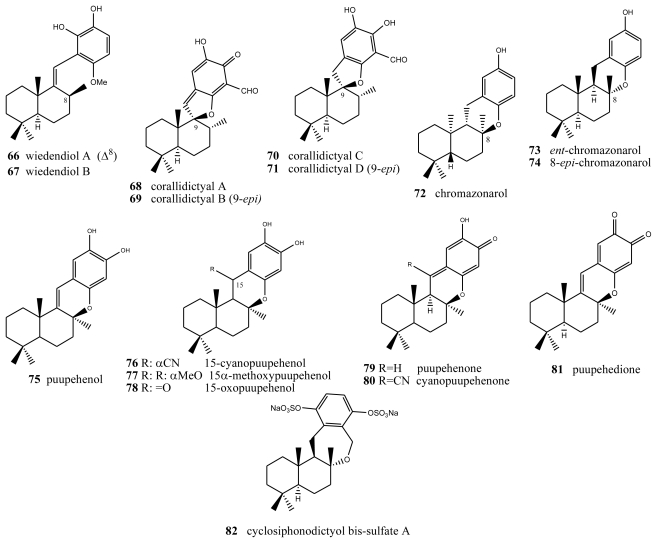
Wiedendiols, corallidictyals, chromazonarols and puupehenols.

Two new bioactive derivatives, wiedendiol A (**66**) and wiedendiol B (**67**) (Figure 4), were isolated from the marine sponge *Xestospongia wiedenmayeri*, collected in the Bahamas [74]. The absolute configuration of these compounds was determined by chemical synthesis of wiedendiol A (**66**), performed from (+)-sclareolide [75]. The CETP-SPA inhibition assays carried out with these compounds revealed an IC_50_ = 5 μM in both cases. Later, the inhibition of CETP was verified using a precipitation method to separate lipoproteins after incubation of HDL radiolabeled with LDL and CETP. In this assay, wiedendiol A (**66**) and B (**67**) had an IC_50_ of 1.0 and 0.6 μM, respectively. Wiedendiol B is a ten-fold stronger inhibitor of cyclooxigenase-2 than the reference compound indomethacine [76].

There are some compounds with a labdane-type decalin that also have a fourth ring, through an oxygen (most times) or carbon bridge between the decalin and the benzo(hydro)quinone ring. For example, structures with a fourth five-membered oxygen ring, in this case spiranic, are the corallidyctals A (**68**), B (**69**), C (**70**) and D (**71**) isolated from the marine sponge *Aka (Siphonodyctio) corallifagum* [77,78]. Both corallidytal A (**68**) and B (**69**) inhibit PKC with an IC_50_ = 28 μM, while assays addressing another cAMP-dependent kinase did not afford inhibition at concentrations of 300 μM, indicating its selectivity. Further, the assays revealed selectivity against the α isoform of PKC [77]. Corallidyctals C (**70**) and D (**71**) were tested in antiproliferative assays using cultures of mouse fibroblasts and activity was linked to the presence of the ortho-hydroquinone moiety [78].

Of the compounds with a fourth six-membered oxygen ring, the first is *ent*-chromazonarol (**73**), isolated from *Dysidea pallescens* [79], whose structure was confirmed by chemical synthesis performed from (−)-sclareol [80]. Its epimer, 8-*epi*chromazonarol (**74**), was isolated from *Smenospongia aurea* [81]. Assays on cytotoxic activity were performed against P-388, A-549. HT-29 and MEL-28 cells, and in all cases an IC_50_ = 15.9 μM was obtained for *ent*-chromazonarol (**73**). Chromazonarol (**72**), isolated from the brown alga, was inactive (IC_50_ > 10 μg/mL) against the KB, Bel-7402, PC-3M, Ketr 3 and MCF-7 human tumor cell lines [82]. An enantioselective cyclisation of 2-(polyprenyl)phenol derivatives to afford polycyclic terpenoids bearing a chroman skeleton such as (−)-chromazonarol by a new artificial cyclase has been described [83].

Two metabolites were obtained from one species of the genus *Verongida*: 15-cyanopuupehenol (**76**) and 15-cyanopuupehenone, whose structure is intimately related to that of puupehenol (**75**) and puupehenone (**80**), the latter isolated from *Stronylophora hartmani*, collected in deep waters off the Bahamas [80,84–87]. Activity assays performed on puupehenone (**79**) revealed cytotoxic activity against different neoplastic lines, with an interesting IC_50_ = 0.5 μM against A-549 and HT-29, and even better inhibition values against the synthesis of DNA and RNA (0.3 and 0.4 μg/mL, respectively). Also, antimicrobial assays provided positive results, especially against *Penicilliium notatum* and *Aspergillus oryzae*. However, in the case of cyanopuupehenone (**78**), only its cytotoxicity against HT-29 human colon carcinoma, with an IC_50_ = 1–2.5 μg/mL, is of interest.

Puuppehenol analogues have been found by studying different species of the genus *Hyrtios*, collected in Hawaii [84] (from which 15α-methoxypuupehenol (**77**), the product of methanol manipulation, was isolated) and New Caledonia (from which 15-oxopuupehenol (**78**) was isolated) [86]. 15α-methoxypuupehenol showed cytotoxic activity (IC_50_ = 6 μg/mL) against KB neoplastic cells. Puupehenone (**80**) was isolated from a sponge of the genus *Verongida*, collected in Hawaii, together with other derivatives of this, among which puupehenione (**81**) [85] is of interest because it is one of the few orthoquinones included in this review. Bioactivity assays carried out on this compound afforded minimum inhibitory concentration values between 1 and 2 μg/mL for all the neoplastic cell lines analyzed. The antimicrobial tests afforded inhibition halos between 10 and 17 mm.

Several routes towards puupehenone-related metabolites have been achieved [88–90]. Puupehenone (**79**) and related compounds were selected in the course of a blind screening for new potential inhibitors of angiogenesis; some of them completely inhibited *in vivo* angiogenesis in the CAM assay at doses equal or lower than 30 nM/egg. They also inhibited the endothelial cell production of urokinase and invasion. The simplicity of their structures and the feasibility of their synthesis make them attractive compounds for further evaluation in the treatment of angiogenesis-related pathologies [91]. Puupenehone analogues from an Indo-Pacific *Hyrtios* sponge showed bioactivity in a soft-agar cytotoxicity test [92].

The only case of compounds with a further six-membered ring is cyclosiphonodictyol bis-sulfate A (**82**), a compound isolated from the marine sponge *Siphonodictyon coralliphagum*. This compound showed inhibitory activity against the binding of [^3^H]-LTB_4_ to human neutrophils, with IC_50_ = 44.5 μM [93].

## 4. Terpenylquinones with a Halimane-Type Decalin Ring

**Figure 5:**
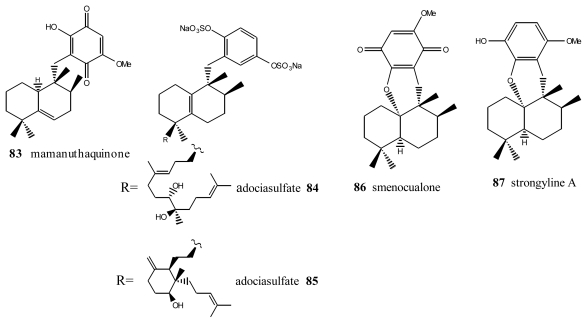
Mamanuthaquinone, smecualone, stronglylin and adociasulfates.

Within this group, the first is mamanuthaquinone (**83**) (Figure 5), a cytotoxic metabolite of *Fasciospongia* sp. collected in the Fiji islands [94]. Activity assays revealed a certain toxicity, especially against HCT-116 human colon carcinoma (IC_50_ = 2 μg/mL). However, *in vitro* anti-HIV activity assays proved to be negative. As indicated above, nakijiquinones J (**17**), M (**18**) and P (**19**) at 1 mM were tested for inhibitory activity against EGFR and HER2 tyrosine kinases. Among them, nakijiquinones P (**19**) exhibited inhibitory activity against EGFR (76% inhibition) [39].

Other compounds with this type of skeleton are the adociasulfates **84** and **85**, isolated from a sponge from the genus *Haliclona*. These two compounds were originally of interest because they were positive in tests studying the inhibition of the ATPase of kinesins, with an IC_50_ = 10 μM for the former and 15 μM for the latter [95]. Smenoqualone (**86**) was isolated from different species of the genus *Smenospongia*, collected from the Gulf of Aden. This compound appears to be related to a product of acid rearrangement of 5*-epi*-isospongiaquinone (**34**), and its structure was determined via its spectroscopic data [96]. The activity assays performed with smenoqualone revealed its inactivity as an antimicrobial, antifungal and cytotoxic agent, suggesting that the presence of a free hydroxyl group on the quinone ring is important for biological activity in this group of compounds. Strongyline A (**87**), a metabolite isolated from the marine sponge *Strongylophora hartmani*, showed cytotoxic activity against P-388 leukemia cells (IC_50_ = 13 μg/mL) and antiviral activity against Influenza PR-8 (IC_50_ = 6.5 μg/mL, IT = 9) [97].

## 5. Other Related Compounds

**Figure 6:**
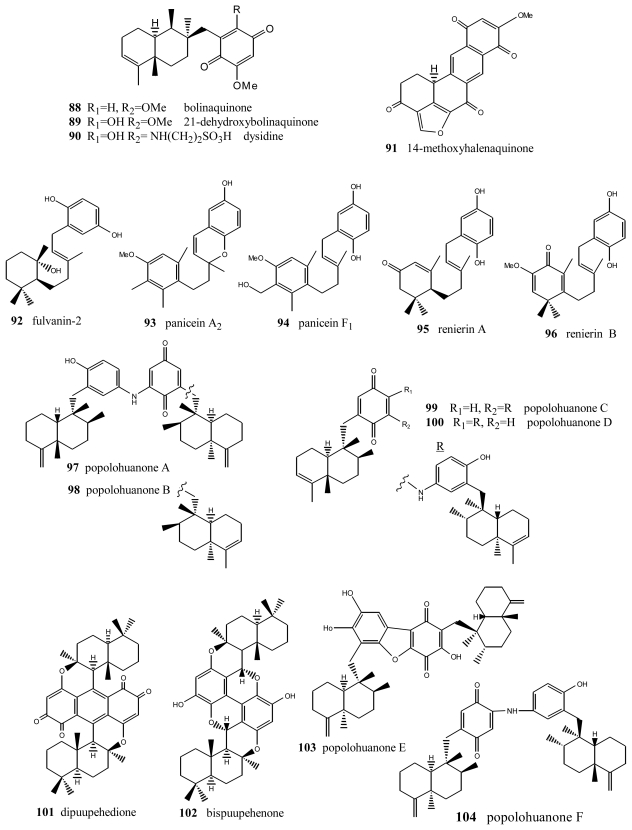
Bolinaquinones, renierins, paniceins, popolohuanones and other compounds.

Bolinaquinone (**88**) (Figure 6) is a cytotoxic sesquiterpene from the genus *Dysidea*, whose quinone moiety is located on an unusual carbon of the decalin [98]. This compound showed cytotoxic activity against HCT-116 human colon carcinoma (IC_50_ = 1.9 μg/mL). The cytotoxicity studies carried out suggest that this compound acts by interfering with or damaging DNA. Dehydroxybolinaquinone (**89**), isolated from the Hainan sponge *Dysidea villosa*, showed moderate PTP1B inhibitory activity and cytotoxicity, with IC_50_ values of 39.5 and 19.5 mM, respectively [99]. The sequiterpene aminoquinone dysidine (**90**) [100], isolated from *Dysidea* sp., had the strongest hPTP1B inhibitory activity, with an IC_50_ value of 6.7 mM [99]. Methoxyhalenaquinone (**91**) from *Xestopolospongia carbonara* is a tyrosine kinase inhibitor [101].

An important number of metabolites with very diverse structures have been isolated from the genus *Reniera*, such as carotenes, alkaloids, diacetylenes and terpenequinones. In particular, four metabolites with a sesquiterpene structure were isolated from the species *Reniera fulva*, with the novelty that they did not have the B ring of the decalin, the most significant compound being fulvanin-2 (**92**) [102]. Additionally, apart from fulvanins, the fractionation of the acetone extract of *Reniera mucosa* afforded another five compounds with analogous structures: paniceins A_2_ (**93**) and F_1_ (**94**), renierins A (**95**) and B (**96**), and *p*-hydroquinone [103]. All the compounds obtained from *Reniera mucosa* were assayed against the P-388, A-549, HT-29 and MEL-28 cell lines with a view to determining their *in vitro* cytotoxicity. Among them, of interest were panicein A_2_ (**93**) (ED_50_ = 5 μg/mL against all the lines assayed) and panicein F_1_ (**94**) (ED_50_ = 5 μg/mL against all cell lines except HT-29), the latter showing medium potency in additional DHFR inhibition tests (ED_50_ = 3 μg/mL).

New terpenylquinones were isolated from different species of the genus *Dysidea*; their structures are formed by two subunits, called popolohuanones. Thus, from a specimen of that genus, collected in Papua New Guinea, popolohuanones A (**97**) and B (**98**) were isolated [104], whereas popolohuanones C (**99**) and D (**100**) were isolated from *Dysidea avara*, collected in the Solomon Islands [64]. Cytotoxicity assays for popolohuanone A (**97**), performed against KB cells at concentrations of 10, 5 and 1 μg/mL, proved to be negative. Identical results were obtained in antimycotic activity assays against *Candida albicans*. Other related compounds were isolated from species of the genus *Hyrtios* (New Caledonia), such as dipuupehenone (**101**) and bispuupehenone (**102**), the latter also found in the species *Hyrtios eubamma* (Tahiti). Bioactivity assays of these compounds unveiled the cytotoxic activity of dipuupehedione (**101**) against KB cells (ED_50_ = 3 μg/mL). Popolohuanone E (**103**), a potent topoisomerase II inhibitor with selective cytotoxicity against the A549 non-small cell human lung cancer cell line, was isolated from *Dysidea* sp. Pohnpei sponges [105], and the biomimetic route to this family of heterocyclic ring systems has been proposed [106]. Popolohuanone A (**96**) and the new dimeric popolohuanone F (**104**) showed DPPH radical scavenging activity, with an IC_50_ value of 35 μM [73].

A biosynthetic pathway leading to several sesquiterpene quinones is suggested [107].

## 6. SAR Studies and Mechanism of Action

Sesquiterpenoid quinones from marine sponges and their semisynthetic derivatives were compared for cytotoxicity on developing eggs of the sea urchin *Strongylocentrotus nudus* and Ehrlich carcinoma cells, and for hemolytic activities on mouse red blood cells. Structure-cytotoxicity studies of several marine sesquiterpenoid quinones and their semisynthetic derivativess on developing eggs of the sea urchin *Strongylocentrotus nudus* and Ehrlich carcinoma cells revealed that the activities of these compounds, with a hydroxyl group at C-20, were higher than their methoxyl and amino groups at this position. Sesquiterpenoid quinones containing a dihydropyran ring had lower activity than non-cyclic compounds. The structure of the terpenoid moieties of the compounds had no significant influence on biological activity. There was a direct correlation between the cytotoxic and hemolytic activities, and the mechanisms of action employed by these compounds against cell membranes have been discussed [108]. Other results from SAR studies appear in the description of the different types of compound.

Regarding the mechanism of action of terpenylquinones, the accumulated data about the biological activity of quinone moieties suggest redox processes and/or Michael-type addition-elimination reactions [31]. Their cytotoxicity has been explained in terms of their ability to undergo redox cycling and the generation of reactive oxygen species, which would damage tumor cells [109–111]. NADH/NADH dehydrogenase reduction of the several terpenylnaphthoquinones increases the rate of oxygen consumption, such rates being higher for quinones with more positive redox potentials. In this process, reactive oxygen species are formed in small amounts, which also correlate with the quinone redox potential. Semiquinone derivatives of these quinones are generated under anaerobic conditions and in the presence of NADH/NADH dehydrogenase. Since this enzymatic system is found in mitochondria, a possible pathway in the cytotoxic activity of these terpenylnaphthoquinones could be interference with or the inhibition of mitochondrial respiration, as reported for other naphthoquinone derivatives, in addition to free radical degradation [29,30]. The results obtained with avarone and avarol supported the mechanism of antitumor action via the reactive oxygen radicals [112,113], but there were also indications of the relevance of arylation of biomolecules, such as proteins [31,114,115].

## 7. Summary

The compounds that appear in this review are meroterpenes, compounds of mixed biogenesis isolated from marine sources and mainly from the following genera: *Dactylospongia*, *Dysidea*, *Euryspongia*, *Fasciospongia*, *Fenestraspongia*, *Haliclona*, *Polifibospongia*, *Siphonodictyon*, *Smenospongia*, *Stelospongia*, *Strongylophora*, *Reniera* and *Xestospongia*. The cytotoxic properties of sesquiterpenequinones or quinols produced by these genera make them viable candidates for continuing the search for analogues with enhanced cytotoxicity, improved selectivity and able to eliminate adverse effects with a view to finding new drugs of marine origin [3,5,19,24–26]. Outstanding among the compounds investigated is 4′-(methylamino)avarone (**58**), with an IC_50_ of 2 μM against melanoma Fem-X cells, and non-cytotoxic to normal lymphocytes [31].
